# Translational rehabilitation strategies for canine medial patellar luxation: insights from human patellofemoral pain syndrome

**DOI:** 10.3389/fvets.2026.1885078

**Published:** 2026-07-08

**Authors:** Ju Young Son, Kyong Kim, You Sung Seo, Seong-Jin Yu, Jeonghyun Choi

**Affiliations:** 1 Department of Rehabilitation Engineering, General Graduate School, Daegu Haany University, Gyeongsan, Republic of Korea; 2 Division of Rehabilitation Therapy, Adventure College, Daegu Haany University, Gyeongsan, Republic of Korea; 3 Advanced Medical Device Research Center, Daegu Haany University, Gyeongsan, Republic of Korea; 4 Center for Neuropsychiatric Research, National Health Research Institutes, Zhunan, Miaoli, Taiwan

**Keywords:** canine rehabilitation, medial patellar luxation, neuromuscular control, patellar tracking, patellofemoral pain syndrome

## Abstract

Medial patellar luxation (MPL) is one of the most common orthopedic disorders in small-breed dogs and is associated with progressive joint degeneration, gait abnormalities, and long-term functional impairment. Despite its high prevalence, current treatment strategies remain primarily focused on structural correction, whereas rehabilitation approaches targeting dynamic patellar stability and functional recovery remain insufficiently established. In particular, rehabilitation concepts addressing muscle function, multi-joint coordination, and neuromuscular regulation have not been systematically integrated into current veterinary rehabilitation paradigms. In human medicine, patellofemoral pain syndrome (PFPS) has been extensively investigated as a disorder characterized by abnormal patellar tracking and dynamic patellar instability associated with altered muscle function and impaired neuromuscular control. Based on these pathophysiological and rehabilitation concepts, this review examined the translational relevance of PFPS-derived rehabilitation strategies to canine MPL. Comparative analysis suggested the presence of partially shared biomechanical characteristics related to patellar tracking and quadriceps function between humans and dogs, despite substantial interspecies differences in gait mechanics and limb loading. Evidence from PFPS rehabilitation studies suggests that interventions emphasizing coordinated muscle function, multi-joint coordination, neuromuscular control, and gait retraining may contribute to restoration of dynamic patellar stability. Accordingly, this review proposes a phase-based, species-adapted translational rehabilitation framework for canine MPL consisting of muscle activation, restoration of multi-joint coordination, neuromuscular regulation, and gait retraining. Although current biomechanical and clinical evidence in dogs remains limited, this framework may provide a conceptual foundation for future function-oriented rehabilitation strategies and prospective validation studies, thereby supporting expansion of canine MPL management beyond structural correction alone toward long-term functional recovery and recurrence prevention.

## 1 Introduction

Medial patellar luxation (MPL) is one of the most commonly encountered orthopedic disorders in small-breed dogs and is recognized as a major cause of hindlimb dysfunction and gait abnormalities. An electronic medical record analysis of small-breed dogs presented to veterinary clinics in Korea reported that musculoskeletal disorders accounted for approximately 6.4% of all clinical visits, with lameness directly associated with gait disturbances observed in 2.9%−4.5% and patellar luxation in 1.4%−1.7% of cases, depending on age group (1). In particular, patellar luxation occurs predominantly in small breeds, including Maltese, Poodles, and Pomeranians (2), with an approximately 12-fold higher prevalence than in large-breed dogs (3, 4). Furthermore, dogs younger than 3 years showed a significantly higher risk of MPL compared with those older than 12 years (OR = 0.4), while females exhibited an approximately 1.3-fold higher prevalence than males (3). Neutering has also been associated with a 2.4–3.1-fold increase in disease risk (3, 5). Collectively, these epidemiological characteristics suggest that MPL should not be considered merely a transient orthopedic abnormality but rather a progressive disorder associated with long-term functional impairment and joint degeneration.

Indeed, MPL is closely associated with cartilage damage, osteoarthritis progression, and secondary orthopedic disorders. In dogs undergoing surgical treatment for MPL, the prevalence of cartilage lesions on the patella and femoral trochlea has been reported to be 47.6% and 54.4%, respectively (6). Moreover, progressive osteoarthritic changes have been reported even after surgical correction (7–9). Cranial cruciate ligament rupture has also been reported in 22%−41% of small-breed dogs and approximately 13% of large-breed dogs with MPL (10), suggesting that structural correction alone may be insufficient to fully prevent functional joint deterioration. These pathological changes may ultimately contribute not only to progressive joint degeneration but also to persistent gait dysfunction, reduced mobility, and impaired physical independence in companion animals. Such long-term functional limitations may substantially compromise quality of life and increase the long-term clinical and economic burden associated with MPL management.

Current treatment strategies for MPL primarily rely on analgesics, anti-inflammatory medications, and surgical correction according to luxation grade (11), whereas rehabilitation approaches targeting postoperative functional recovery and recurrence prevention remain underdeveloped (12, 13). A review of rehabilitation studies following MPL surgery revealed that most currently available evidence consists of case reports or small case series, while prospective comparative studies remain scarce. Although some studies have reported improvements in recovery and functional outcomes following rehabilitation interventions, the overall evidence base remains limited and predominantly consists of lower-level evidence. Notably, the only prospective comparative study identified in this review reported no significant differences between intervention groups, whereas most remaining evidence is derived from case series and case reports (Table 1). Consequently, substantial limitations in study design, sample size, and overall evidence quality restrict definitive conclusions regarding the efficacy of rehabilitation interventions for canine MPL. Furthermore, existing rehabilitation protocols have largely focused on pain reduction and maintenance of joint range of motion (ROM), whereas rehabilitation strategies targeting dynamic patellar stability, muscle function, and neuromuscular regulation remain poorly defined in veterinary clinical practice. As a result, postoperative functional recovery, restoration of dynamic patellar stability, and recurrence prevention remain insufficiently addressed in current clinical management.

**Table 1:** Summary of rehabilitation studies in canine MPL.

Study	Evidence level	Study design	Experimental group	Control group	Results
Akaraphutiporn et al. (12)	Level 2	Prospective clinical trial	Regular home exercise + ES	Regular home exercise (PROM, Standing, Walking etc.)	Lameness and pain score ↓
			Regular home exercise + LASER		
					TMC ↓ Function & QOL ↑
					N.S. (Exp. vs. Cont.)
Rajabian et al. (13)	Level 4	Case series	NMES + US + MT + PROM + FE	–	TMC, ROM, TUG ↑
					Lameness ↓
Karasiewicz et al. (14)	Level 5	Case study	MT + PROM + FE + Hydrotherapy	–	TMC, ROM, QOL ↑
					Lameness ↓
Kang et al. (15)	Level 5	Retrospective study, Case study	Heat + LASER, FE	Time-dependent recovery	Recovery time ↓

The table summarizes currently available clinical studies evaluating rehabilitation interventions following canine MPL, including exercise therapy, NMES, US, MT, PROM, FE, hydrotherapy, and laser therapy. Across studies, rehabilitation interventions were generally associated with improvements in lameness, pain, joint mobility, functional outcomes, and quality of life; however, the only prospective comparative trial identified in this review reported no significant differences between intervention groups. Although study designs and intervention protocols varied considerably, these findings suggest the potential clinical value of multimodal rehabilitation approaches for improving postoperative recovery and functional stabilization in dogs with MPL while highlighting the need for higher-quality prospective studies. Evidence levels were assigned according to the Oxford Center for Evidence-Based Medicine (OCEBM) Levels of Evidence. The prospective clinical trial was classified as Level 2 evidence, the case series as Level 4 evidence, and the case study-based reports as Level 5 evidence. ES, electrical stimulation; LASER, light amplification by stimulated emission of radiation; PROM, passive range of motion; TMC, thigh muscle circumference; QOL, quality of life; N.S., no significant difference; NMES, neuromuscular electrical stimulation; US, therapeutic ultrasound; MT, manual therapy; FE, functional exercises; TUG, timed up and go.

Despite the high prevalence of MPL and the increasing clinical adoption of rehabilitation in veterinary medicine, current rehabilitation approaches remain largely symptom-oriented and lack a mechanistic framework targeting dynamic patellar stability and neuromuscular function. In particular, rehabilitation strategies addressing coordinated muscle activation, multi-joint coordination, and neuromuscular regulation during movement have not been sufficiently integrated into current veterinary rehabilitation paradigms. To date, no review has systematically examined whether biomechanical and neuromuscular rehabilitation concepts established in human patellofemoral disorders can be selectively translated into canine MPL rehabilitation. This unresolved gap highlights the need for a translational, function-oriented framework capable of complementing conventional structure-based treatment strategies.

Normal patellar tracking is maintained through the interaction between structural alignment and neuromuscular regulation during movement. In the canine stifle joint, the patella follows a consistent tracking pathway throughout flexion and extension (16, 17), and this dynamic stability is considered important for maintaining normal gait. In this context, dynamic patellar stability refers to the ability to maintain appropriate patellar tracking during movement through the coordinated interaction of musculoskeletal alignment, muscle function, and neuromuscular regulation. However, current MPL treatment strategies remain predominantly focused on structural realignment, while functional mechanisms involved in the restoration of dynamic patellar stability have received comparatively limited attention in veterinary rehabilitation research (11, 18, 19).

In this context, patellofemoral pain syndrome (PFPS) in humans may provide a particularly relevant translational reference because it represents one of the most extensively investigated conditions involving abnormal patellar tracking, quadriceps dysfunction, and neuromuscular impairment. Previous studies have suggested that impaired coordination of the quadriceps muscle group, altered neuromuscular regulation, and deficient lower extremity control contribute to abnormal patellar mechanics and dynamic instability (20, 21). Importantly, contemporary PFPS rehabilitation has progressively shifted from isolated strengthening approaches toward integrated rehabilitation strategies emphasizing neuromuscular control, multi-joint coordination, and gait retraining rather than isolated muscle strengthening alone. These conceptual advances may provide a valuable translational framework for exploring functional rehabilitation approaches in canine MPL.

Although humans and dogs differ substantially in anatomical structure and gait pattern, both species exhibit several comparable biomechanical characteristics in that the patella functions as a critical component of quadriceps force transmission and follows a dynamic tracking pathway during joint flexion and extension. These shared biomechanical characteristics suggest that selected rehabilitation principles derived from human PFPS may provide a species-adapted translational framework for canine MPL rehabilitation. Nevertheless, because dogs are quadrupeds with fundamentally different limb alignment and weight-bearing mechanics from humans, direct protocol transfer of human rehabilitation strategies remains inherently limited. Therefore, rehabilitation strategies for canine MPL should not simply replicate human rehabilitation models but instead be reconstructed using a species-adapted translational approach that considers both biomechanical similarities and species-specific differences.

Accordingly, this reviewaims to examine the translational relevance of biomechanical and rehabilitation concepts derived from human PFPS for canine MPL. Rather than advocating direct transfer of human rehabilitation protocols, this review proposes a species-adapted conceptual framework based on selectively translatable biomechanical and neuromuscular principles associated with the restoration of dynamic patellar stability. In particular, this review focuses on the roles of muscle function, multi-joint coordination, and neuromuscular regulation in functional recovery following MPL, and proposes a phase-based translational rehabilitation framework as a hypothesis-generating conceptual model incorporating muscle activation, restoration of multi-joint coordination, neuromuscular regulation, and gait retraining. Importantly, the proposed framework is intended to generate testable hypotheses and guide future translational research. Rather than representing a validated clinical rehabilitation strategy, it should be viewed as a conceptual model whose translational applicability remains to be established through prospective biomechanical and clinical studies before broader clinical adoption. By integrating biomechanical reinterpretation with neuromuscular rehabilitation perspectives, this review aims to provide a conceptual foundation for future translational veterinary rehabilitation research and support the development of function-oriented clinical strategies for canine MPL.

## 2 Literature search strategy

A narrative literature review was conducted to identify studies relevant to canine MPL, human PFPS, patellar biomechanics, neuromuscular control, and rehabilitation strategies. Electronic databases including PubMed, Scopus, and Web of Science were searched for articles published up to April 2026.

The search strategy combined terms related to canine MPL and human PFPS, including “medial patellar luxation”, “patellar luxation”, “canine rehabilitation”, “patellofemoral pain syndrome”, “patellar tracking”, “quadriceps function”, “neuromuscular control”, “gait retraining”, “biomechanics”, and “rehabilitation”. Additional relevant articles were identified through manual screening of reference lists from key publications.

Studies were included if they investigated patellar biomechanics, neuromuscular function, gait characteristics, rehabilitation interventions, or functional outcomes in canine MPL or human PFPS. Clinical trials, observational studies, biomechanical investigations, systematic reviews, and relevant narrative reviews published in English or Korean were considered eligible when deemed relevant to the objectives of this review.

Given the limited availability of rehabilitation studies specifically addressing canine MPL, evidence from the human PFPS literature was additionally reviewed to identify biomechanical and neuromuscular concepts potentially applicable to canine rehabilitation from a translational perspective. The objective of this review was not to perform a systematic evidence synthesis, but rather to examine the conceptual and translational relevance of PFPS-derived rehabilitation principles to canine MPL. Accordingly, formal systematic review procedures, including risk-of-bias assessment and quantitative meta-analysis, were not performed.

## 3 Comparison of the patellofemoral joint between humans and dogs: Applicability and limitations of translational rehabilitation strategies

Although the human and canine patellofemoral joints differ in anatomical structure and gait pattern, they exhibit several comparable biomechanical characteristics with respect to the functional role and kinematic behavior of the patella. In both species, the patella functions as a biomechanical lever that facilitates the transmission of quadriceps-generated force to the tibia and follows a defined tracking pathway during joint flexion and extension (17, 22–24). In humans, the patella has been reported to enhance the mechanical efficiency of quadriceps force transmission by increasing the quadriceps moment arm by approximately 15%−30% (22). Similarly, in dogs, the patella serves as a key biomechanical lever in stifle extension mechanics; however, its dynamic stability depends on precise patellar tracking along the trochlear groove during movement. Quantitative evidence from canine studies suggests that patellar tracking is highly sensitive to structural alignment. Finite element modeling demonstrated that patellar medial displacement begins when the anatomical lateral distal femoral angle (aLDFA) reaches or exceeds 103°, at which point the contact reaction force between the patella and trochlear groove approaches zero (19). Furthermore, *in vivo* kinematic analyses have demonstrated that stifle joint pathology alters patellar tracking trajectories relative to normative patterns observed in healthy dogs (25). Although comparable biomechanical roles have been proposed in dogs, quantitative studies evaluating these mechanisms during quadrupedal locomotion remain limited. These findings suggest that although the patella serves broadly comparable biomechanical functions across species, the magnitude and biomechanical manifestation of these effects may differ according to species-specific locomotor mechanics.

These biomechanical abnormalities may contribute to clinically measurable functional impairment. Dogs with Grade III MPL have been reported to exhibit significantly reduced stifle ROM prior to surgical correction (26), while objective force plate analyses have identified persistent ground reaction force asymmetry in surgically treated dogs even when visual gait assessment appeared clinically normal (8). Collectively, these findings suggest that patellar function in dogs, as in humans, should not be regarded as merely a passive structural role but rather as a dynamic biomechanical process susceptible to disruption by malalignment and altered neuromuscular regulation. This perspective further supports the need for rehabilitation strategies targeting dynamic patellar stability beyond structural correction alone.

Studies on human PFPS have consistently demonstrated that dynamic patellar stability is closely associated with functional regulation of the quadriceps muscle group rather than being determined solely by static structural alignment (20, 21, 27). In particular, imbalance between the vastus medialis oblique (VMO) and vastus lateralis (VL) has been proposed as an important mechanism contributing to abnormal lateral patellar displacement. In patients with PFPS exhibiting patellar maltracking, the VL:VM activation ratio showed a significant correlation with patellar tilt (*r*^2^ = 0.72, *p* = 0.002). Furthermore, increased VL:VM activation ratio was directly associated with delayed VM activation (*r*^2^ = 0.76, *p* = 0.001) (21). These findings suggest that neuromuscular imbalance may substantially influence dynamic patellar tracking behavior and contribute to functional patellofemoral instability in PFPS, supporting the concept that dynamic patellar stability is influenced not only by structural alignment but also by the coordinated neuromuscular regulation of the quadriceps muscle group.

Similarly, in the canine stifle joint, the patella moves within the femoral trochlear groove throughout flexion and extension, and the stability of this tracking pathway plays an important role in maintaining normal gait. Dogs with patellar luxation exhibit abnormal patellar tracking and functional gait disturbances (16, 17), suggesting that patellar stability may also be influenced by neuromuscular regulatory mechanisms in addition to structural alignment. However, no canine electromyographic studies have validated VMO-VL activation timing alterations in dogs with MPL. Moreover, direct investigations examining the relationship between VMO/VL activation patterns and patellar tracking in dogs are currently lacking (28–30). This represents a major knowledge gap in the translational application of muscle-based rehabilitation strategies derived from human PFPS to canine MPL. Accordingly, although dynamic patellar stability and functional quadriceps regulation may represent translationally relevant biomechanical concepts across species, VMO-VL activation timing mechanisms should not be directly translated to canine MPL without further canine-specific investigation. Their application to canine rehabilitation requires cautious interpretation and species-specific electromyographic and kinematic validation.

Importantly, the translational applicability of these rehabilitation principles must be interpreted within the context of species-specific biomechanical differences. Humans are upright bipeds and primarily load the lower extremities during locomotion, whereas dogs are quadrupeds with body weight distributed across four limbs (31). Force plate analyses in clinically normal dogs have demonstrated that the hindlimbs support a peak vertical force of approximately 65% of body weight during trotting, compared with approximately 107% in the forelimbs (32). These findings indicate that the mechanical loading environment of the canine stifle differs substantially from that experienced by the human knee during upright single-limb stance. Consequently, the direction and magnitude of joint loading, as well as the biomechanical demands throughout the gait cycle, differ fundamentally between the two species. In addition, differences exist in femoral and tibial alignment, soft tissue organization, and the conceptual applicability of alignment indices such as the Q-angle (18, 31). In dogs, breed-specific skeletal deformities frequently represent major pathophysiological contributors to patellar luxation (11, 18), which limits the extent to which muscle-centered mechanisms can independently explain disease progression or guide rehabilitation strategies, as has been proposed in human PFPS.

Taken together, these similarities and differences suggest that rehabilitation principles derived from the human patellofemoral joint should not be viewed as universally transferable models for canine MPL but rather as selectively translatable approaches applicable to specific biomechanical and neuromuscular components. Dynamic patellar stability-related factors, including patellar tracking and functional quadriceps regulation, may represent relevant rehabilitation targets in dogs. In contrast, rehabilitation concepts primarily based on Q-angle correction, upright load redistribution, and human-specific hip–knee coordination models may have limited applicability because of fundamental anatomical and locomotor differences between species. Table 2 summarizes the proposed translational applicability of key PFPS-derived rehabilitation concepts to canine MPL by categorizing them as readily translatable, partially translatable, or not directly translatable.

**Table 2:** Selective translational applicability of rehabilitation concepts derived from human PFPS to canine MPL.

PFPS-derived concept	Translational applicability	Rationale
Dynamic patellar stability	Readily translatable	Appropriate patellar tracking during movement contributes to dynamic patellar stability in both human PFPS and canine MPL
Neuromuscular control of patellar stability	Partially translatable	Neuromuscular control likely contributes to patellar stability in both species, although canine-specific mechanistic evidence remains limited
Multi-joint coordination and gait retraining	Partially translatable	Functional movement coordination is relevant across species, although implementation must be adapted to quadrupedal locomotion
Q-angle-based rehabilitation concepts	Not directly translatable	The Q-angle concept has limited direct biomechanical applicability to canine stifle biomechanics
Dynamic knee valgus correction and upright load redistribution strategies	Not directly translatable	These concepts are primarily based on human bipedal locomotion and weight-bearing mechanics

Selected rehabilitation concepts derived from human PFPS were classified according to their proposed translational applicability to canine MPL as readily translatable, partially translatable, or not directly translatable. Classification was based on the authors' synthesis of currently available evidence regarding shared biomechanical principles, neuromuscular mechanisms, and species-specific differences in anatomy and locomotor mechanics. This framework is intended to illustrate the selective and species-adapted nature of translational rehabilitation strategies rather than to imply direct clinical transfer of human rehabilitation protocols to dogs. PFPS, patellofemoral pain syndrome; MPL, medial patellar luxation.

**Figure 1:**
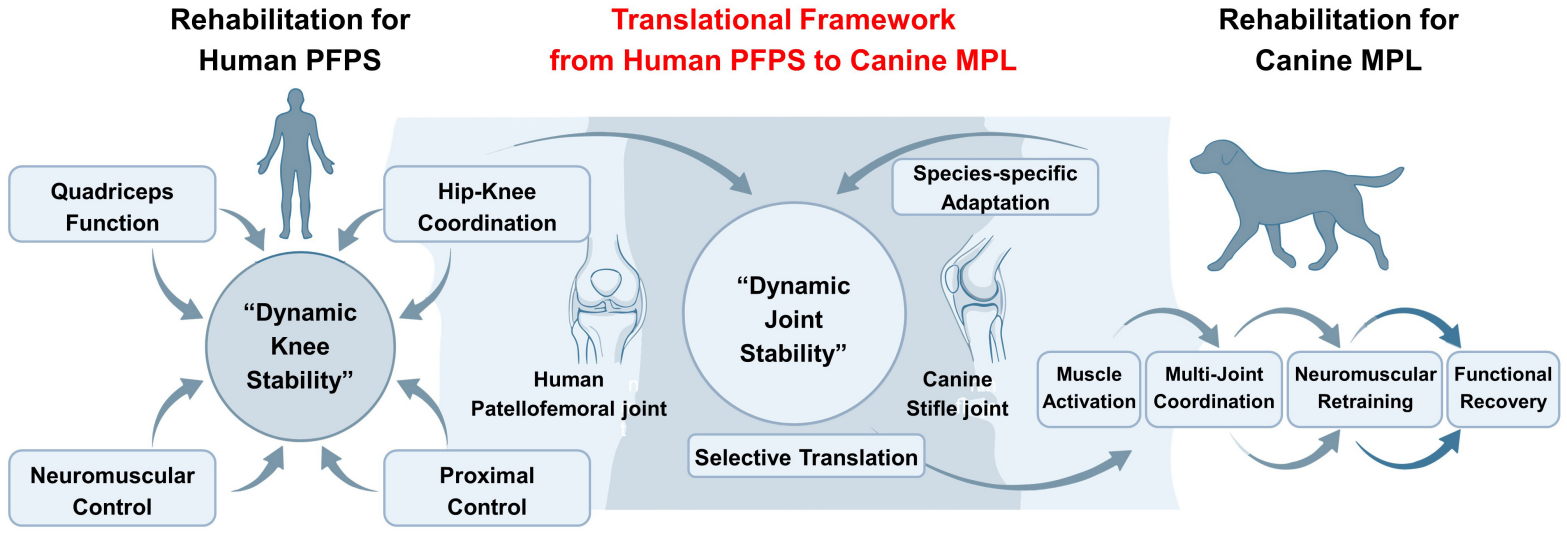
Mechanistic interaction underlying dynamic patellar instability in human PFPS and its translational relevance to canine MPL. This schematic illustrates the proposed mechanistic interaction among structural malalignment, neuromuscular dysfunction, kinetic chain impairment, and abnormal patellar tracking underlying dynamic patellar instability in patellofemoral pain syndrome (PFPS). Structural contributors, including altered alignment and soft tissue imbalance, interact with neuromuscular factors such as altered quadriceps function, proprioceptive impairment, and fatigue susceptibility, as well as proximal kinetic chain dysfunction involving altered hip and lower extremity mechanics. These interacting factors contribute to increased patellofemoral joint stress, pain, functional locomotor impairment, and progressive dynamic instability during movement. The figure further highlights the potential translational relevance of selected biomechanical and neuromuscular concepts to canine medial patellar luxation (MPL), including abnormal patellar tracking, dynamic instability, gait asymmetry, and locomotor dysfunction. Rather than representing a direct transfer of human rehabilitation models, the schematic emphasizes selectively translatable principles that may support the development of species-adapted rehabilitation strategies targeting restoration of dynamic patellar stability and functional locomotor recovery in dogs with MPL.

Despite substantial interspecies differences in anatomy, limb loading, and locomotor mechanics, both PFPS and MPL involve disruption of normal patellar tracking, altered quadriceps function, locomotor impairment, and reduced dynamic joint stability. In both conditions, abnormal patellar mechanics are associated with functional deficits that cannot be fully explained by structural alignment alone. These shared biomechanical characteristics provide the primary rationale for exploring the translational applicability of rehabilitation concepts derived from PFPS to canine MPL. Accordingly, rehabilitation strategies for canine MPL should emphasize species-adapted integration of dynamic neuromuscular control mechanisms rather than direct replication of human rehabilitation protocols. The proposed framework therefore focuses on broadly conserved principles of dynamic patellar stability and neuromuscular regulation while accounting for species-specific anatomical and locomotor differences. Such an approach may provide a conceptual foundation for the future development of function-oriented rehabilitation strategies tailored specifically to canine MPL. Figure 1 summarizes the proposed mechanistic interaction among abnormal patellar tracking, neuromuscular dysfunction, and kinetic chain impairment underlying dynamic patellar instability in PFPS, together with their potential translational relevance to canine MPL rehabilitation.

## 4 Pathophysiology of patellofemoral pain syndrome: Interaction between abnormal patellar tracking and neuromuscular factors

PFPS is increasingly recognized as a disorder of dynamic instability arising from the interaction between abnormal patellar tracking and the mechanical and neuromuscular factors that influence it rather than as a condition caused by a single etiological factor. Abnormal patellar tracking reduces the contact area within the patellofemoral joint, thereby increasing focal contact pressure and shear stress under the same joint loading conditions. Farrokhi and Keyak (37). reported that patients with PFPS exhibited approximately 17%−36% increases in mean and peak hydrostatic pressure and 35%−60% increases in octahedral shear stress within patellofemoral cartilage compared with healthy controls. These alterations in the mechanical environment are considered to be important contributors to pain, functional impairment, and progressive joint degeneration (38, 39). These findings suggest that abnormal patellar tracking may contribute not only to pain generation but also to progressive deterioration of joint mechanics during movement.

Abnormal patellar tracking is influenced not only by structural alignment factors but also by imbalances in surrounding soft tissues and muscle function. Increased Q-angle, laxity of the medial retinaculum, excessive tension of the lateral retinaculum, and increased iliotibial band tension may generate static lateral forces on the patella, thereby promoting lateral displacement and contributing to symptom severity (40–42). Merican and Amis demonstrated that increasing iliotibial band tension to 60 N and 90 N resulted in approximately 0.8–1.4 mm of lateral patellar displacement and 0.7°-1.5° increases in lateral patellar tilt within the mid-flexion range (43). However, static alignment factors alone appear insufficient to fully explain dynamic patellar instability during movement. In a prospective cohort study following 1,319 individuals over three years, no significant difference in Q-angle was identified between individuals who developed PFPS and those who did not (*F* = 0.22, *p* = 0.64) (44). Similarly, studies involving novice runners and general populations reported that Q-angle did not significantly predict PFPS or running-related injuries (45, 46). A recent systematic review likewise failed to support the hypothesis that Q-angle represents an independent risk factor for PFPS (42). Collectively, these findings support the concept that dynamic patellar instability in PFPS is more strongly associated with impaired neuromuscular regulation during movement than with static structural factors alone.

In addition to structural and alignment-related factors, patients with PFPS frequently exhibit a wide range of neuromuscular impairments, including reduced quadriceps strength, impaired proprioception, altered excitability of the monosynaptic reflex pathway, and increased susceptibility to fatigue (47–49). Such individuals have also demonstrated reduced knee joint proprioceptive accuracy and earlier onset of quadriceps fatigue during functional activities compared with healthy controls (49, 50). Meta-analytic evidence further suggests that hip abductor and external rotator strength are reduced in individuals with PFPS compared with healthy controls, indicating that patellofemoral instability may reflect proximal kinetic chain dysfunction as well as local quadriceps impairment (51, 52). These neuromuscular deficits may function not only as consequences of abnormal patellar tracking but also as factors that perpetuate the condition, thereby contributing to chronicity through interactions between structural alignment abnormalities and impaired neuromuscular control.

Among these factors, functional imbalance within the quadriceps muscle group has been proposed as an important contributor to abnormal patellar tracking and dynamic instability. Delayed activation of the VMO relative to the VL has frequently been observed in patients with PFPS (27, 53), and altered activation timing has been associated with increased risk of subsequent PFPS development (54). Specifically, delayed VMO activation demonstrated significant correlations with lateral patellar tilt (*r*^2^ = 0.89, *p* < 0.001) and bisect offset (*r*^2^ = 0.75, *p* = 0.012) (20). In a prospective cohort study, Van Tiggelen et al. reported that a VMO–VL activation delay greater than −0.67 ms was associated with increased risk of PFPS development (AUC = 0.68). Such neuromuscular control delays may impair dynamic patellar stabilization during the early phase of knee movement and thereby reinforcing abnormal tracking patterns (21). Furthermore, studies demonstrating improvements in pain and function following rehabilitation interventions that restored synchronous or earlier VMO activation relative to VL support the notion that restoration of neuromuscular regulation may contribute to recovery of dynamic patellar stability (53). However, because comparable electromyographic evidence is currently unavailable in dogs with MPL, these findings should not be directly translated to canine MPL and require further canine-specific investigation before translational application. Therefore, rehabilitation strategies for canine MPL should focus primarily on overall quadriceps function and dynamic patellar stability rather than selective modulation of individual quadriceps components.

Taken together, the pathophysiology of PFPS cannot be sufficiently explained by structural malalignment alone. Rather, PFPS should be understood as a disorder of dynamic patellar instability arising from the interaction among abnormal patellar tracking, imbalances in muscle activation, impaired proprioception, and other neuromuscular dysfunctions, including fatigue susceptibility. Accordingly, abnormal patellar tracking may be interpreted as a functional manifestation of these interacting biomechanical and neuromuscular factors. These concepts suggest that rehabilitation strategies targeting dynamic patellar instability should extend beyond correction of static alignment and should instead emphasize restoration of coordinated muscle activation, neuromuscular control, and functional movement patterns during locomotion.

## 5 Evidence-based rehabilitation strategies for patellofemoral pain syndrome: Key interventions for selective translational application

Based on the pathophysiological mechanisms described above, rehabilitation strategies for PFPS have evolved toward enhancing muscle function and neuromuscular regulation in order to restore dynamic patellar stability. A meta-analysis comparing exercise interventions with conservative treatment approaches (12 randomized controlled trials, *n* = 601–719) demonstrated that exercise-based rehabilitation significantly improved pain (mean difference = −0.8, 95% CI −1.23 to −0.37) and functional outcomes (mean difference = 1.3, 95% CI −1.21 to 3.81) (55). These findings suggest that exercise-based rehabilitation may provide clinically meaningful improvements in both pain and functional recovery in PFPS. Furthermore, they support the concept that rehabilitation strategies targeting movement-related neuromuscular dysfunction may improve functional patellar stability beyond passive symptom management. On the basis of such evidence, exercise therapy is currently regarded as a primary rehabilitation strategy for PFPS.

Quadriceps strengthening exercises represent the most traditional and widely applied intervention in PFPS rehabilitation. Early rehabilitation models emphasized selective strengthening of the VMO as a strategy for dynamic medial stabilization of the patella (56, 57), and selective VMO retraining subsequently became a major rehabilitation strategy in PFPS management. However, subsequent controlled studies and systematic reviews consistently reported no significant differences in pain reduction or functional improvement between selective VMO strengthening and generalized quadriceps strengthening programs (33, 58). These findings suggest that restoration of coordinated neuromuscular regulation across the quadriceps muscle group may be more important for maintaining dynamic patellar stability than selective strengthening of an individual quadriceps component.

More recently, rehabilitation strategies emphasizing coordination between the hip and knee joints have emerged as central components of PFPS management. Weakness of the hip musculature, particularly the abductors and external rotators, has been associated with increased dynamic knee valgus and altered lower extremity mechanics, thereby increasing lateral displacement forces acting on the patella (59, 60). Biomechanical studies have further demonstrated that individuals with PFPS may exhibit increased hip adduction and internal rotation during functional tasks, suggesting that these proximal kinematic alterations may contribute to abnormal patellar loading (59, 61). Consequently, hip–knee coordination exercises have gained attention as interventions designed to improve integrated lower extremity kinetic chain function. In a randomized controlled trial comparing quadriceps-focused and gluteal-focused exercise programs over a 12-week period, both groups demonstrated improvements of more than 7 points on the Anterior Knee Pain Scale (AKPS) (7.6 vs. 7.0 points, respectively), with statistically equivalent improvements in pain and function (34). Furthermore, studies reporting larger effect sizes for pain reduction and functional improvement in groups receiving combined hip and knee interventions compared with knee-focused interventions alone (35) support the concept that dynamic patellar instability may arise from impaired multi-joint coordination and kinetic chain dysfunction rather than pathology confined to a single joint.

Neuromuscular training extends beyond isolated muscle strengthening and aims to improve movement coordination, balance, contraction timing, and sensorimotor integration during functional tasks. In a randomized controlled trial involving patients with PFPS, the addition of 4 weeks of core and lower extremity neuromuscular training to conventional physical therapy resulted in significantly greater improvements in pain (*p* = 0.035), function (*p* = 0.039), and dynamic balance measures, including posteromedial reach performance (*p* = 0.016), compared with conventional therapy alone (36). Importantly, all observed improvements exceeded the minimum clinically important difference (MCID). These findings suggest that rehabilitation approaches targeting sensorimotor integration and dynamic movement control may enhance functional stability during locomotor tasks and may provide superior clinical outcomes compared with isolated muscle strengthening alone.

Taken together, rehabilitation strategies with demonstrated efficacy in PFPS are not limited to selective strengthening of individual muscles such as the VMO, but instead focus on improving coordinated regulation of the quadriceps and hip musculature, restoring coordinated multi-joint movement, and enhancing neuromuscular control throughout the lower extremity (Table 3). Because these intervention components target movement-based neuromuscular regulation relevant to dynamic patellar stability, they may represent selectively translatable rehabilitation principles for canine MPL based on partially shared biomechanical characteristics between the human and canine stifle joint.

**Table 3:** Summary of representative rehabilitation studies in human PFPS relevant to translational application in canine MPL rehabilitation.

Study	Subjects	Intervention	Main results	Clinical implication
Cowan et al. (27)	Adults with PFPS, *n* = 65, 6-week PT	McConnell based program 6-week PT	VMO onset shifted	Improvement of VMO/VL timing is associated with rehabilitation of PFPS
			(BT: VL → VMO, AT-CC: VMO = VL, AT-EC: VMO → )	
			Pain ↓, Funtion ↑	
Kooiker et al. (33)	PFPS patients in 7 RCTs	PT-guided quadriceps strengthening vs. Advice/minimal intervention	Quadriceps strengthening: pain ↓, Function ↑	Supervised quadriceps strengthening should be a core first-line treatment for PFPS
Hansen et al. (34)	Adults with PFPS, n=200	QE *vs* HE program 12-week	AKPS change: QE 7.6 vs. HE 7.0	Clinicians can choose hip- or quadriceps-focused programs; Both yield similar symptom and function gains
			(No MCID)	
			Difference 0.6 (95% CI −2.0 to 3.2): Equivalent improvements	
Nascimento et al. (35)	PFPS patients in 14 RCTs	Hip + Knee strengthening vs. Knee strengthening vs. Placebo	(i) Hip + Knee vs. Knee strengthening: – Muscle power: N.S. – Pain: Hip + Knee ↓ (Score: −3.3) – Function: Hip + Knee ↑ (Score: 0.6) (ii) Hip + Knee strengthening vs. placebo – Muscle power: N.S. – Pain: Hip + Knee ↓ (Score: −1.5)	Adding hip strengthening to knee programs provides superior pain and activity outcomes vs. knee-only strengthening
Motealleh et al. (36)	Women with unilateral PFPS, *n* = 28	Core NMT+PT vs. PT 4-week	Pain: core NMT <PT (*p* = 0.035)	Core NMT (timing, coordination, balance) enhances effects of conventional PFPS exercise therapy
			Kujala: core NMT > PT (*p* = 0.039)	
			Step-down: core NMT > PT (*p* = 0.027)	
			Y-balance (Posteromedial): core NMT > PT (*p* = 0.016)	

The table summarizes key clinical studies investigating quadriceps strengthening, hip-focused exercise, combined hip–knee rehabilitation, and NMT in individuals with PFPS. Reported outcomes generally demonstrate improvements in pain, functional performance, and movement coordination, highlighting the importance of dynamic joint stability and neuromuscular control. These findings provide a conceptual translational rationale for incorporating muscle activation, multi-joint coordination, and gait-oriented neuromuscular rehabilitation strategies into canine MPL rehabilitation frameworks. PFPS; Patellofemoral pain syndrome, MPL; Medial patellar luxation, PT; Physical therapy, MaConnell based program; Patellar taping + Progressive functional retraining of VMO using dual channel sEMG biofeedback + Gluteal strengthening exercise and stretching of soft tissue structure; BT Before therapy; AT-CC, After therapy-Concentric contraction; AT-EC, After therapy-Eccentric contraction; QE, Quadriceps strengthening exercise; HE, hip strengthening exercise; MCID, Minimal clinically important change; N.S., no significant difference; NMT, neuromuscular training.

## 6 Translational applicability of rehabilitation strategies for canine MPL: Proposal of a translational rehabilitation framework

Current veterinary rehabilitation following MPL surgery commonly incorporates passive range of motion (PROM) exercises, therapeutic exercise, hydrotherapy, neuromuscular electrical stimulation, therapeutic ultrasound, manual therapy, and laser therapy. As summarized in Table 1, these interventions have generally been associated with improvements in pain, lameness, joint mobility, and functional recovery. However, most currently available studies consist of case reports and small case series, and rehabilitation approaches primarily focus on pain management and restoration of joint function, whereas rehabilitation strategies specifically targeting dynamic patellar stability, neuromuscular regulation, and movement coordination remain insufficiently developed. These limitations provide an important rationale for exploring translational rehabilitation concepts aimed at improving dynamic patellar stability, neuromuscular regulation, and movement coordination in canine MPL.

As discussed above, rehabilitation strategies with demonstrated efficacy in human PFPS primarily target restoration of dynamic patellar stability through improvement of muscle function, multi-joint coordination, and neuromuscular regulation. Although these principles may be selectively applicable to canine MPL, direct protocol transfer remains limited because of substantial interspecies differences in anatomical structure and gait mechanics. Therefore, translational application of evidence-based human rehabilitation strategies to canine MPL requires a species-adapted approach that preserves shared biomechanical principles while accounting for the unique characteristics of the canine stifle joint and quadrupedal locomotion.

**Figure 2:**
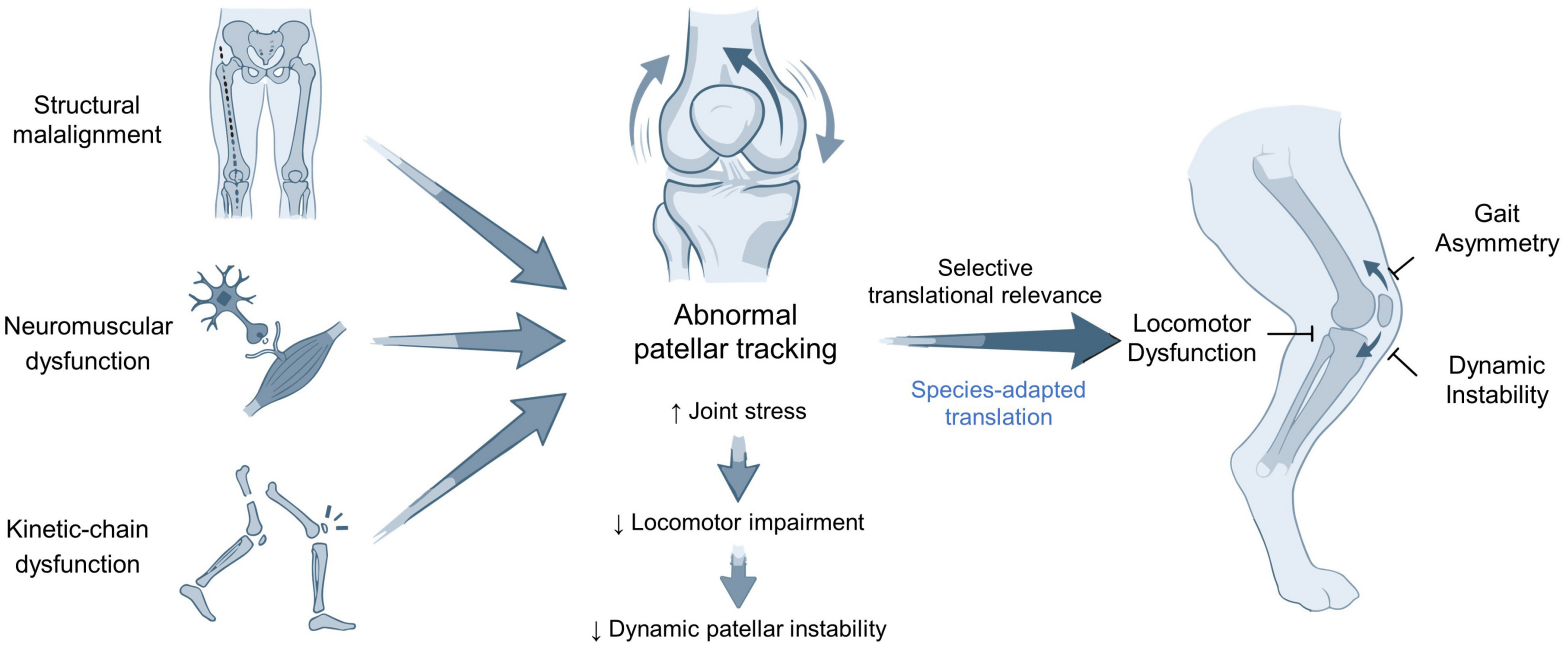
Species-adapted translational rehabilitation framework for canine MPL derived from human PFPS concepts. The schematic illustrates a species-adapted translational rehabilitation framework in which biomechanical and neuromuscular principles derived from human patellofemoral pain syndrome (PFPS), including quadriceps function, hip–knee coordination, proximal control, and neuromuscular regulation, are selectively translated to canine medial patellar luxation (MPL) rehabilitation. Rather than representing a direct clinical transfer, the framework emphasizes selective translation based on partially shared biomechanical principles while accounting for species-specific differences between bipedal and quadrupedal locomotion. Sequential rehabilitation components, including muscle activation, restoration of multi-joint coordination, neuromuscular regulation, and gait retraining, are integrated to support restoration of dynamic patellar stability, locomotor function, and long-term functional recovery in dogs with MPL.

The rehabilitation framework proposed in this review is conceptually based on clinical guidelines for PFPS rehabilitation that emphasize combined hip–knee strengthening, restoration of multi-joint coordination, and improvement of neuromuscular control as key rehabilitation components (62, 63). Because the biomechanical and neuromuscular mechanisms underlying canine MPL have not yet been fully elucidated, the proposed framework is intended to provide a conceptual basis for future investigation rather than a prescriptive clinical protocol. The framework was developed primarily in the context of postoperative rehabilitation following surgical correction of Grade II–IV MPL, where restoration of dynamic patellar stability, muscle function, and gait mechanics constitutes a major rehabilitation objective. Although selected principles may also have relevance to conservatively managed cases, their applicability in non-surgical management remains to be established. Accordingly, the three-phase structure presented below should be viewed as a translational model designed to facilitate hypothesis generation and prospective validation studies in canine rehabilitation. To selectively translate these principles to canine MPL, the rehabilitation process is conceptually structured into three sequential phases: (1) muscle activation and establishment of foundational dynamic patellar stability, (2) recovery of multi-joint coordination, and (3) neuromuscular control and gait retraining. Although each phase shares the overarching objective of restoring dynamic patellar stability, progression should not be determined by fixed timelines but instead adjusted according to individual pain level, weight-bearing capacity, gait characteristics, and overall functional recovery. Objective gait and kinetic assessments, such as weight-bearing symmetry and ground reaction force analysis, may serve as measurable indicators for monitoring functional progression and guiding phase advancement during rehabilitation in dogs with MPL. Future studies may also evaluate objective progression criteria for phase advancement. Examples of candidate thresholds could include achievement of ≥80% hindlimb weight-bearing symmetry, completion of 10 consecutive sit to stand repetitions without observable lameness, or restoration of predefined gait symmetry metrics. However, such criteria remain hypothetical and require prospective validation before clinical implementation. Figure 2 summarizes the conceptual translational rehabilitation framework proposed for selective application of PFPS-derived rehabilitation principles to canine MPL.

The initial phase focuses on restoring foundational muscle activation while minimizing excessive joint loading. In dogs with MPL, pain and structural instability may lead to reduced activation of the quadriceps and surrounding hip musculature. This phenomenon may conceptually resemble arthrogenic muscle inhibition observed in humans, in which nociceptive input from the joint suppresses quadriceps neuromuscular activation (64). Because impaired muscle activation may contribute to reduced dynamic patellar stability during the early recovery phase, the initial rehabilitation phase should emphasize low-intensity muscle activation, sensory feedback, and redistribution of weight-bearing patterns to reduce abnormal loading and establish foundational dynamic stability (65). Progressive restoration of symmetrical hindlimb loading may further contribute to reducing abnormal patellofemoral mechanical stress during early functional recovery. Representative interventions during this phase may include PROM exercises, assisted weight-shifting activities, and controlled weight-bearing exercises aimed at facilitating early muscle activation and restoration of foundational dynamic patellar stability.

During the functional recovery phase, rehabilitation should progress beyond isolated joint-focused interventions toward integrated retraining of the entire hindlimb kinetic chain, including the hip, stifle, and tarsal joints. Considering that abnormal patellar tracking may arise from deficits in multi-joint coordination, restoration of proximal hindlimb muscle function may contribute to stabilization of hindlimb mechanics and reduction of abnormal displacement forces acting on the patella (35, 66, 67). In addition, progressive weight-bearing modulation may facilitate gradual adaptation of patellofemoral mechanical loading during functional recovery and contribute to improved dynamic stability (63, 65). Accordingly, this phase should focus not only on restoration of muscle strength but also on coordinated recovery of interlimb weight distribution, interjoint coordination, and functional hindlimb integration during gait. Examples of Phase 2 interventions may include sit to stand exercises and cavaletti rail training, which may promote coordinated multi-joint movement, progressive functional loading, and recovery of dynamic hindlimb function.

The final phase aims to restore integrated neuromuscular regulation and re-establish normal gait patterns (36, 68). At this stage, the primary objective shifts from isolated muscle strengthening to integrated neuromuscular control, including movement coordination, contraction timing, balance, and proprioceptive regulation. Studies in human PFPS have suggested that isolated muscle strengthening alone may be insufficient to fully restore normal movement patterns such as excessive hip adduction, internal rotation, and pelvic drop, highlighting the need for movement pattern retraining (69). Furthermore, gait retraining studies have reported up to approximately 81% reduction in pain following 6 weeks of feedback-based gait retraining, with sustained improvements during follow-up (68), while a 12-week intervention reduced patellofemoral joint stress by approximately 13.3% (70). However, evidence from PFPS rehabilitation studies remains heterogeneous, and the approximately 81% pain reduction reported in this study may represent an outlier rather than a typical response observed across gait-retraining interventions. Similarly, dogs with MPL commonly exhibit abnormal gait characteristics, including skipping gait, hindlimb weight-bearing asymmetry, and irregular stride patterns, which may persist even after postoperative pain reduction (8, 26). Objective kinetic analyses have further shown that ground reaction force asymmetry may persist despite apparent clinical improvement on visual gait assessment (8). Therefore, restoration of more symmetrical and coordinated movement patterns through gait retraining may represent an important component of the final rehabilitation phase. In dogs, gait retraining may be interpreted as a species-adapted strategy aimed at restoring symmetrical hindlimb loading, stride regularity, and coordinated limb movement during locomotion. Representative interventions during this phase may include underwater treadmill training and obstacle-navigation exercises designed to enhance neuromuscular control, locomotor adaptability, gait symmetry, and movement coordination.

Taken together, rehabilitation for canine MPL should not be regarded solely as muscle strengthening or postoperative supportive care (65), but rather be conceptualized as an integrated process progressing from muscle activation to restoration of multi-joint coordination and ultimately to neuromuscular regulation and gait retraining. The proposed framework is not intended to replace structural correction-based treatment, but rather to provide complementary rehabilitation principles aimed at improving dynamic patellar stability and functional recovery. Nevertheless, because this framework represents a conceptual translational model derived from evidence in human PFPS, future prospective biomechanical, kinematic, and clinical studies incorporating objective functional outcome measures are warranted to validate the translational applicability of this framework in dogs with MPL.

## 7 Limitations and future directions

In this review, we proposed a translational rehabilitation framework for canine MPL based on evidence accumulated from studies of human PFPS. However, this approach remains primarily conceptual and hypothesis-generating, and several important limitations should be considered before broad clinical application. Accordingly, the proposed framework should be interpreted as a preliminary translational model intended to guide future research, and its translational applicability remains to be established through prospective biomechanical and clinical studies. In addition, the framework was developed primarily in the context of postoperative rehabilitation following surgical correction of Grade II–IV MPL, and its applicability to dogs managed conservatively or to dogs with mild Grade I luxation remains to be established.

First, interspecies differences between humans and dogs represent a major limitation to directly transferring rehabilitation strategies. Humans are upright bipeds, whereas dogs are quadrupeds with fundamentally different patterns of weight distribution, joint alignment, and biomechanical demands during locomotion (16, 71). These differences influence the direction and magnitude of forces acting on the patellofemoral joint, as well as the mechanical characteristics of the gait cycle. Consequently, interventions shown to be effective in humans may not necessarily produce comparable outcomes in dogs. Therefore, the proposed framework should not be interpreted as a direct transfer of human rehabilitation strategies, but rather as a species-adapted translational model that integrates shared principles of dynamic patellar stability to the anatomical and functional characteristics of canine locomotion.

Second, the limited availability of quantitative biomechanical data related to canine MPL represents another major constraint. In human PFPS research, pathophysiological mechanisms and treatment effects have been extensively investigated using quantitative indicators such as patellar tracking patterns, muscle activation timing, and intra-articular pressure distribution. In contrast, quantitative biomechanical studies in dogs remain scarce, particularly with respect to *in vivo* three-dimensional analysis of patellofemoral joint kinematics (19). Although MPL is one of the most common causes of hindlimb lameness in dogs, the biomechanical and neuromuscular mechanisms underlying its pathophysiology have not yet been fully elucidated (11). This limited mechanistic understanding hinders validation of the theoretical basis of the rehabilitation strategies proposed in this review.

Third, clinical evidence supporting rehabilitation interventions for canine MPL remains insufficient. Most available studies are limited to case reports or small-scale clinical investigations, and studies evaluating postoperative rehabilitation protocols frequently involve single cases or limited sample sizes (11, 14). Prospective comparative studies evaluating specific rehabilitation modalities have only recently emerged (12). Furthermore, standardized functional assessment tools and objective outcome measures for evaluating rehabilitation efficacy have not yet been sufficiently established. These limitations hinder both the generalizability of rehabilitation outcomes and the development of standardized clinical protocols.

To address these limitations, stepwise translational investigations are required. First, biomechanical studies that quantitatively examine the relationship between dynamic patellar tracking and activation patterns of the quadriceps and proximal hindlimb musculature in dogs with MPL are needed. In particular, integration of three-dimensional kinematic analysis, electromyographic assessment, and ground reaction force analysis may provide objective insights into dynamic patellar instability and compensatory gait strategies associated with MPL. Such studies would contribute substantially to validating the mechanistic basis of the proposed rehabilitation framework. Establishment of objective biomechanical and neuromuscular outcome measures may further facilitate phase-specific rehabilitation monitoring and translational validation in dogs with MPL.

In addition, prospective clinical studies using standardized assessment tools are necessary. Future investigations should incorporate comprehensive multidimensional outcome measures, including pain, ROM, muscle function, weight-bearing asymmetry, gait characteristics, recurrence rates, and quality-of-life indicators. Comparative studies evaluating structural correction alone vs. structural correction combined with postoperative rehabilitation, as well as comparisons among different rehabilitation protocols, are also warranted. Furthermore, real-time gait analysis and movement pattern analysis using wearable sensors or image-based motion analysis technologies may facilitate continuous functional monitoring and the development of individualized rehabilitation strategies tailored to locomotor recovery status.

In conclusion, the translational framework proposed in this review provides a conceptual foundation for the development of translational rehabilitation strategies targeting canine MPL. However, establishment of clinically applicable rehabilitation protocols for canine MPL will require quantitative biomechanical investigations and high-quality clinical studies that appropriately account for interspecies differences. Such future research may contribute to shifting current treatment paradigms beyond structural correction alone toward a more integrated, function-oriented approach to canine MPL management that incorporates biomechanical and neuromuscular perspectives.

## 8 Conclusion

In this review, we highlighted that although canine MPL is a highly prevalent disorder associated with progressive joint degeneration, current treatment strategies remain largely focused on structural correction, whereas rehabilitation approaches targeting functional recovery remain comparatively underdeveloped. In particular, muscle function and neuromuscular control, both of which contribute to dynamic patellar stability, may play important roles in functional recovery in dogs with MPL, yet these factors have not been sufficiently incorporated into current rehabilitation strategies.

Research on human PFPS has demonstrated that abnormal patellar tracking is not merely a problem of structural malalignment, but rather a manifestation of dynamic patellar instability arising from interactions among muscle function, neuromuscular regulation, and movement-related biomechanical factors. Based on these pathophysiological and rehabilitation concepts, the present review examined the potential translational relevance of rehabilitation strategies derived from human PFPS to canine MPL.

Accordingly, we proposed a phase-based translational rehabilitation framework consisting of muscle activation, restoration of multi-joint coordination, neuromuscular regulation, and gait retraining. This framework is centered on the restoration of dynamic patellar stability and provides a conceptual foundation for the development of function-oriented rehabilitation strategies intended to complement conventional structural correction-based treatment approaches. Importantly, this framework does not represent a direct transfer of human rehabilitation models, but rather a species-adapted translational strategy that considers both partially shared biomechanical principles and species-specific differences between humans and dogs.

Although quantitative biomechanical investigations and high-quality clinical evidence for canine MPL rehabilitation remain limited, the framework proposed in this review may serve as a conceptual basis for the development and future clinical validation of function-oriented neuromuscular rehabilitation protocols supported by objective biomechanical and locomotor outcome measures. Furthermore, the accumulation of prospective studies incorporating kinematic, electromyographic, and gait analysis may ultimately expand current canine MPL management paradigms beyond postoperative supportive care toward a more integrated, function-oriented rehabilitation approach for long-term functional recovery and recurrence prevention. Importantly, the proposed framework is intended to guide future translational research, and its translational applicability should be established through prospective biomechanical and clinical studies before broader implementation in veterinary rehabilitation practice.
